# 704 Hydroxocobalamin Is Not Associated with Methemoglobinemia in Patients with Inhalation Injury and Suspected Cyanide Toxicity

**DOI:** 10.1093/jbcr/irae036.249

**Published:** 2024-04-17

**Authors:** Eloise Stanton, Sarah Wang, Kenneth Han, Fiona Garlich, Haig A Yenikomshian, Justin Gillenwater

**Affiliations:** Keck School of Medicine of USC, Los Angeles, CA; Keck SOM of USC, Los Angeles, CA; Los Angeles General Medical Regional Burn Center, Los Angeles, CA; LA General Medical Center, Los Angeles, CA; University of Southern California, Los Angeles, CA; Keck Medicine of USC, Los Angeles, CA; Keck School of Medicine of USC, Los Angeles, CA; Keck SOM of USC, Los Angeles, CA; Los Angeles General Medical Regional Burn Center, Los Angeles, CA; LA General Medical Center, Los Angeles, CA; University of Southern California, Los Angeles, CA; Keck Medicine of USC, Los Angeles, CA; Keck School of Medicine of USC, Los Angeles, CA; Keck SOM of USC, Los Angeles, CA; Los Angeles General Medical Regional Burn Center, Los Angeles, CA; LA General Medical Center, Los Angeles, CA; University of Southern California, Los Angeles, CA; Keck Medicine of USC, Los Angeles, CA; Keck School of Medicine of USC, Los Angeles, CA; Keck SOM of USC, Los Angeles, CA; Los Angeles General Medical Regional Burn Center, Los Angeles, CA; LA General Medical Center, Los Angeles, CA; University of Southern California, Los Angeles, CA; Keck Medicine of USC, Los Angeles, CA; Keck School of Medicine of USC, Los Angeles, CA; Keck SOM of USC, Los Angeles, CA; Los Angeles General Medical Regional Burn Center, Los Angeles, CA; LA General Medical Center, Los Angeles, CA; University of Southern California, Los Angeles, CA; Keck Medicine of USC, Los Angeles, CA; Keck School of Medicine of USC, Los Angeles, CA; Keck SOM of USC, Los Angeles, CA; Los Angeles General Medical Regional Burn Center, Los Angeles, CA; LA General Medical Center, Los Angeles, CA; University of Southern California, Los Angeles, CA; Keck Medicine of USC, Los Angeles, CA

## Abstract

**Introduction:**

Cyanide poisoning is a serious threat to burn patients, especially those exposed to smoke from fires, often leading to cyanide toxicity and cardiovascular collapse. Cyanokit, a widely used hydroxocobalamin product able to convert cyanide molecules into safe compounds, is known for its potential to avoid methemoglobinemia, a side effect of other treatments. Concerns about cyanokit's safety, especially in burn patients, have arisen due to recent cases with elevated methemoglobin levels. This study investigates methemoglobin levels in burn patients treated with Cyanokit, aiming to enhance our understanding of cyanide antidote therapy complexities, ultimately leading to safer treatment strategies.

**Methods:**

A retrospective study was conducted of burn patients admitted to a single institution from 2013-2023 with inhalation injury treated with cyanokit for suspected cyanide toxicity. We also analyzed a control cohort of similar patients with inhalation injury not treated with cyanokit. We analyzed changes and peaks in methemoglobin, lactate levels, kidney function tests, ventilator days, % total body surface area (TBSA), medications and dressings, and mortality.

**Results:**

In the study, 36 patients with suspected inhalation injury were treated with cyanokit, compared to 32 patients with inhalation injury who weren't treated with cyanokit. Baseline characteristics showed no statistically significant differences between the groups, including age, gender, BMI, and %TBSA. No significant differences were found in initial, final, peak, or change in methemoglobin levels, initial lactate levels, mortality, Baux score, kidney function tests, ventilator days, surgeries, or use of medications/treatments (e.g., Silvadene dressings, Vitamin C) between the two groups (Table 1). When controlling for covariates, multiple linear regression analysis (age, gender, and %TBSA) indicated that cyanokit administration was not significantly associated with changes in methemoglobin or mortality.

**Conclusions:**

Contrary to our initial hypothesis, our study did not find significant harm or benefit of cyanokit in burn patients during the acute phase. Methemoglobin, lactate levels, mortality, and kidney function remained largely unchanged. This study's limitations, particularly the rarity of inhalation injury cases with concern for cyanide toxicity, warrant consideration. Further research is required to comprehensively elucidate the impact of cyanokit administration on burn patients' outcomes.

**Applicability of Research to Practice:**

Given the high stakes involved in treating cyanide poisoning promptly, our study holds significance for refining treatment strategies and enhancing patient safety. This insight is vital for medical practitioners making critical decisions in managing cyanide poisoning in burn patients, while also underlining the need for further research to fully understand the broader impact of cyanokit administration in this context.

